# Haloperidol Induced Cell Cycle Arrest and Apoptosis in Glioblastoma Cells

**DOI:** 10.3390/biomedicines8120595

**Published:** 2020-12-11

**Authors:** Fotios Papadopoulos, Rafaela Isihou, George A. Alexiou, Thomas Tsalios, Evrysthenis Vartholomatos, Georgios S. Markopoulos, Chrissa Sioka, Pericles Tsekeris, Athanasios P. Kyritsis, Vasiliki Galani

**Affiliations:** 1 Neurosurgical Institute, Medical School, University of Ioannina, 45500 Ioannina, Greece; fotispap08@gmail.com (F.P.); rafisy94@hotmail.gr (R.I.); eyrys.varth@gmail.com (E.V.); csioka@yahoo.com (C.S.); THKYRITS@uoi.gr (A.P.K.); 2 Department of Anatomy-Histology-Embryology, Medical School, University of Ioannina, 45500 Ioannina, Greece; tomtsalios7@gmail.com (T.T.); vgalani@uoi.gr (V.G.); 3 Laboratory of Biology, School of Medicine, University of Ioannina, 45500 Ioannina, Greece; geomarkop@gmail.com; 4 Department of Radiation Oncology, University of Ioannina, 45500 Ioannina, Greece; ptsekeri@cc.uoi.gr

**Keywords:** haloperidol, antipsychotic, apoptosis, glioblastoma cells

## Abstract

Although several antipsychotic drugs have been shown to possess anticancer activities, haloperidol, a “first-generation” antipsychotic drug, has not been extensively evaluated for potential antineoplastic properties. The aim of this study was to investigate the antitumoral effects of haloperidol in glioblastoma (GBM) U87, U251 and T98 cell lines, and the effects of combined treatment with temozolomide (TMZ) and/or radiotherapy, using 4 Gy of irradiation. The viability and proliferation of the cells were evaluated with trypan blue exclusion assay and 3-(4,5-dimethylthiazol-2-yl)-2,5-diphenyltetrazolium bromide (MTT) assay. Apoptosis, using the annexin-propidium iodide (PI), and cell cycle, cluster of differentiation (CD) expression and caspase-8 activation were measured using flow cytometry. Treatment with haloperidol significantly reduced cell viability in U87, U251 and T98 GBM cell lines. Haloperidol induced apoptosis in a dose-dependent manner, inhibited cell migration and produced an alteration in the expression of CD24/CD44. The additional effect of haloperidol, combined with temozolomide and radiation therapy, increased tumor cell death. Haloperidol was observed to induce apoptosis and to increase caspase-8 activation. In conclusion, haloperidol may represent an innovative strategy for the treatment of GBM and further studies are warranted in glioma xenograft models and other malignancies.

## 1. Introduction

Glioblastoma (GBM) is the most aggressive glial brain tumor in adults, with a median life expectancy of 15 months, despite gross total excision, radiotherapy and temozolomide (TMZ)-based chemotherapy [1]. GBM shows a high degree of phenotypic and genetic heterogeneity, which contribute to treatment failure and disease recurrence [2]. In addition, intrinsic brain tumors are “protected” by the presence of the normal blood–brain barrier (BBB), which allows only the selective permeation of certain low molecular weight substances and blocks several chemotherapeutic agents [3]. Drug repositioning has attracted interest because it can speed up the therapeutic developmental process. Antineoplastic properties against various cancers have been demonstrated by some antipsychotics, including risperidone, an inhibitor of the serotonin receptor 7, which when used to treat schizophrenia in a patient with GBM, resulted in survival of 6.5 years [4]. Pimozide, another antipsychotic drug, was found to impair GBM cell growth and stem cell survival [5], and several other antipsychotic drugs, including thioridazine, remoxipride and MRJF4, a novel haloperidol metabolite II prodrug, have been tested in glioma cell-based experiments [6,7,8,9,10]. Haloperidol is a dopamine D2 receptor antagonist that was developed in the 1950s and belongs to the typical or ‘’first generation’’ antipsychotics [11]. TMZ has been shown to work synergistically with dopamine D2 receptor antagonists to inhibit the proliferation of GBM U251 and A172 cells [9]. U251 and U87 cells are sensitive to TMZ, whereas T98 show inherited resistance [12]. To the best of our knowledge, no previous study has evaluated the combined effect of radiotherapy with haloperidol. In this study, we investigated the antitumoral effects of haloperidol in U251, U87 and T98 GBM cell lines, both alone and in combination with TMZ and/or radiotherapy.

## 2. Experimental Section

### 2.1. Cell Lines and Treatment Conditions

The human glioma cell lines U87 and U251 were obtained from Dr W.K. Alfred Yung (Department of Neuro-Oncology, M.D. Anderson Cancer Center, Houston, TX, USA) and T98 were obtained from ATCC (Manassas, VA, USA). All cell lines were cultured in Dulbecco’s modified Eagle’s medium (DMEM, Gibco BRL, Life Technologies, Grand Island, NY, USA) supplemented with 10% fetal bovine serum (FBS) and 1% penicillin–streptomycin (Gibco BRL). All cell lines were incubated in a humidified atmosphere containing 5% CO_2_ at 37 °C. Before every experiment, haloperidol (Sigma Aldrich, St. Louis, MO, USA) and TMZ (Sigma Aldrich, St. Louis, MO, USA) were diluted in dimethyl sulfoxide (DMSO) from stock solution (1 mM) to the final concentration with culture medium. Cultures of malignant glioma cells were treated with haloperidol or TMZ, or both, with and without radiotherapy.

### 2.2. Viability Assay

For the viability assays, glioma cell lines were treated with haloperidol in concentrations of 5, 10, 20, 50 and 100 μM for the U87 and U251 cell lines, and in concentrations of 10, 20, 40, 80 and 160 μΜ for the T98 cell line. Cell viability was evaluated by the trypan blue exclusion assay and 3-(4,5-dimethylthiazol-2-yl)-2,5-diphenyltetrazolium bromide (MTT, Sigma Life Sciences) assay [13,14,15]. Approximately 5000 cells were seeded in 96-well plates, and after 24 h were exposed to escalating concentrations of haloperidol for another 72 h without medium change. At 72 h, MTT was added. The amount of MTT-formazan was determined at 570 nm. Trypan blue exclusion assay was performed. Both methods were carried out in triplicate at least three times, and the results were expressed as the mean of the three. The cell cultures were observed every day via light microscopy.

### 2.3. Flow Cytometric Analysis of Apoptosis, DNA Cell Cycle, Caspase-8 and Cluster of Differentiation (CD) Expression

Cells (10^4^) were treated with haloperidol at various concentrations. As a negative control, cells treated with equal volumes of plain culture medium were used. All cell samples were run in triplicate in at least three independent experiments. For the DNA cell cycle analysis, the cells were harvested after incubation with trypsin, centrifuged, washed with phosphate-buffered saline solution (PBS), and incubated with propidium iodide (PI) working solution (50 µg/mL PI, 20 mg/mL RNase A, and 0.1% Triton X-100) at 37 °C in the dark for 20 min. Data from the PI fluorescence was collected to a total count of 10,000 nuclei, using a flow cytometer (FACScalibur, BD Biosciences, San Jose, CA, USA). Using the CellQuest software program (BD Biosciences) the cell cycle fractions G0/G1, S, G2/M, and sub-G0/G1 were analyzed as described previously [13]. The DNA cell cycle was investigated with PI, as described previously in detail [13,14], using the annexin V–FITC/PI apoptosis detection kit I (BD Bioscience Pharmingen, San Diego, CA, USA). The activity of caspase-8 was quantified with the Fluorescein Active Caspase-8 Staining Kit (Abnova, Taiwan) as described previously [15]. For the assessment of the cluster of differentiation (CD) expression, specifically CD24/CD44, we used the FITC mouse anti-human CD24 (ML5), PE mouse anti-human CD24 (ML5), and FITC mouse anti-human CD44 (Leu-44) (all from BD Pharmingen) as described previously in detail [16]. Approximately 10,000 cells were seeded in 24-well plates and after 24 h were exposed to a concentration of 100 μΜ of haloperidol, for another 72 h. The cells were then dissociated by trypsinization, washed twice with PBS and in order to block Fc receptors, incubated with 10% human serum for 20 min on ice. The corresponding antibodies were then added and the cells were analyzed by a flow cytometer (FACScalibur, Becton Dickinson, San Jose, CA, USA). Using Cell Quest software program (BD Biosciences) the results were determined for each histogram as the geometric mean peak of fluorescence intensity.

### 2.4. Wound Healing Assay

U87 and T98 cells were treated with haloperidol at concentrations of IC50 and twice the IC50 values when the cells reached 70–80% confluence. The monolayer cells were scratched using a 200 μL pipette tip at the bottom of the well and were then cultivated under standard conditions (DMEM supplemented with 10% FBS). The migration distance at 0, 48 and 72 h after scratching was estimated for each cell line.

### 2.5. Combination Treatment with Haloperidol, TMZ and Radiation

Cultures of malignant glioma cells were treated with either haloperidol, TMZ, or a combination of haloperidol and TMZ, with or without radiotherapy. U87 and T98 cells were cultured in 24-well plates and after 24 h were treated with haloperidol and/or TMZ, then after a further 24 h irradiated at 4 Gy as previously described [17]. Viability was evaluated using a trypan blue exclusion assay and MTT assay.

### 2.6. Statistical Analysis

The data were expressed as the mean ± standard deviation (SD). Two-way ANOVA with post hoc Tukey test for multiple comparisons was used to investigate the significance of differences between the results of different experimental conditions. Differences were considered significant at *p* values < 0.05.

## 3. Results

### 3.1. Sensitivity of GBM Cells to Haloperidol and IC50 Calculation

**Figure 1:**
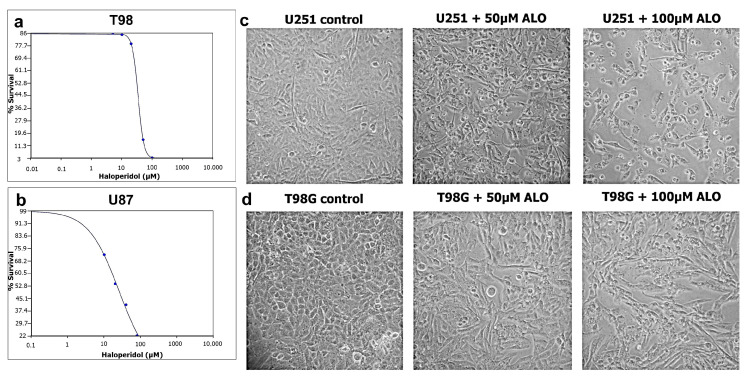
Viability of glioma cells following haloperidol (ALO) treatment. Cell viability was assessed by the trypan blue exclusion test and MTT in T98 (**a**) and U87 (**b**) glioma cells. Viability tests were performed 72 h after haloperidol treatment. Values shown are the means and standard deviations from the three independent experiments. Values are normalized to non-treated cells (* *p* < 0.05 vs. control). (**c**) Microscopy (100×) observation of the U251 and T98 (**d**) cell lines after treatment with haloperidol (50 and 100 μΜ) for 72 h.

To determine the anti-glioma activity of haloperidol in GBM cells, U87, U251 and T98 cells were incubated with increasing haloperidol concentrations for 72 h. All the cell lines were sensitive to treatment with haloperidol in a dose-dependent manner. Using the trypan blue exclusion assay and MTT, the IC50 value of reduced viability for haloperidol was 23 μM in U87 cells, 35 μM in T98 and 38 μM in U251 cells (Figure 1a,b). On microscopic observation, treatment with increasing haloperidol concentrations produced changes in the morphology of U251 and T98 cells, such as nuclear fragmentation and cell shrinkage, indicating cell death, probably by apoptosis (Figure 1c,d).

### 3.2. Haloperidol Induced G2/M Cell Cycle Arrest and Appearance of subG0/G1 Peak

**Figure 2:**
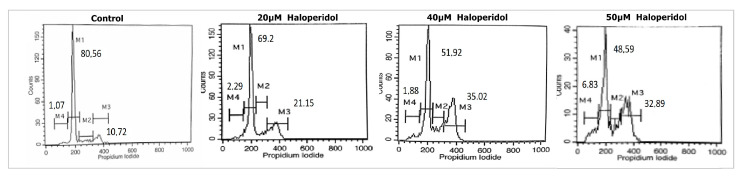
Cell-cycle distribution assessed by flow cytometry in U87 glioblastoma cells. Approximately 10,000 cells were seeded in 24-well plates and after 24 h were exposed to escalating concentrations of haloperidol for another 72 h. At 72 h, the cells were stained by propidium iodide and the DNA content was evaluated.

To investigate the cell cycle events underlying the observed growth inhibitory effects, we evaluated the effects of haloperidol on cell cycle progression in the U87 cell line. Cell cultures were treated with IC50 and twice the IC50 values of haloperidol for 72 h. Haloperidol induced a G2/M cell cycle arrest and an increase in the percentage of cells in sub G0/G1 in a dose dependent manner, suggesting the induction of apoptosis (Figure 2, Table 1).

### 3.3. Haloperidol Induced Apoptosis and Increased Caspase-8 Activation in GBM Cells

**Figure 3:**
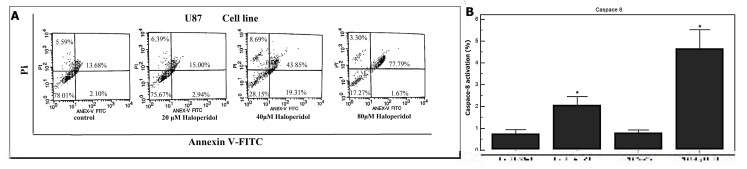
The effect of haloperidol on the induction of apoptosis in U87 glioblastoma cells. (**A**) Flow cytometry was used to analyze the early and late phases of apoptotic cells by annexin V-FITC/PI staining. This assay quantifies live viable cells (left bottom quadrant), apoptotic cells (right bottom and right top quadrants) and necrotic cells (left top quadrant). (**B**) U251 and T98 glioblastoma cells (10,000 cells) were incubated in 24-well plates, and after 24 h, haloperidol (40 μΜ) was added. After an additional 72 h period, caspase-8 was measured. Significant increase (*p* < 0.05) in caspase-8 activation was demonstrated in both cell lines.

To clarify whether the death observed under microscopy and in cell cycle analysis (increase in subG0/G1) was due to necrosis or to programmed cell death (i.e., apoptosis), the ability of haloperidol to promote an apoptotic effect in the U87 cell line was assessed. First, we analyzed whether haloperidol induced apoptotic phenomena during treatment for 12 and 24 h at IC50 values (Figure S1). Following cytometry analysis, we found that haloperidol did not induce subG0/G1 cell population, however, a cell cycle arrest in G2/M was observed. Based on the aforementioned results, we analyzed the cell for apoptotic phenomena at a later time frame. Apoptotic cells were measured by flow cytometry after 72 h treatment with haloperidol, using PI/annexin V staining. The results showed that haloperidol treatment significantly increased the percentage of U87 cells undergoing apoptosis. Relative to the control cells, the percentage of apoptosis in U87 cells treated with haloperidol for 48 h increased from 13.68 to 77.79%, while the percentage of living cells decreased from 78.01 to 17.27% (Figure 3A). Caspase-8 activation was investigated in U251 and T98 cells. The results showed that haloperidol significantly increased caspase-8 activation in both cell lines (Figure 3B).

### 3.4. Haloperidol Induced Changes in CD Expression in U251 and T98 Cells

**Figure 4:**
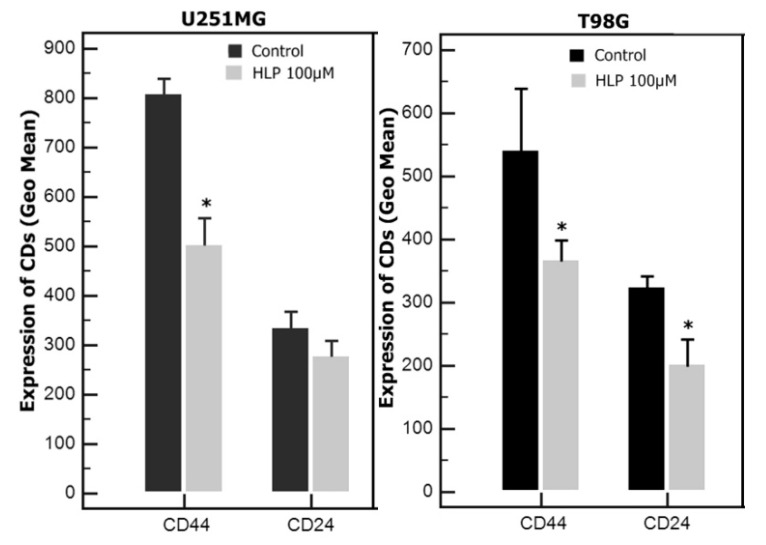
Flow cytometry analysis for the expression of the cluster of differentiation (CD). CD44 and CD24 expression in U251 and T98 glioblastoma cells after haloperidol treatment (HLP). Significant differences (*p* < 0.05) are marked with an asterisk.

To investigate the expression of CD markers associated with migration, invasion and metastasis in U251 and T98 cells, 10,000 cells were seeded and after 24 h exposed to 100 μΜ haloperidol. Significant decrease in the expression of CD44 was observed in both cell lines. Decrease in the expression of CD24 was observed in both cell lines, but the decrease was statistically significant only in T98 (Figure 4).

### 3.5. Haloperidol Inhibited Cell Migration

**Figure 5:**
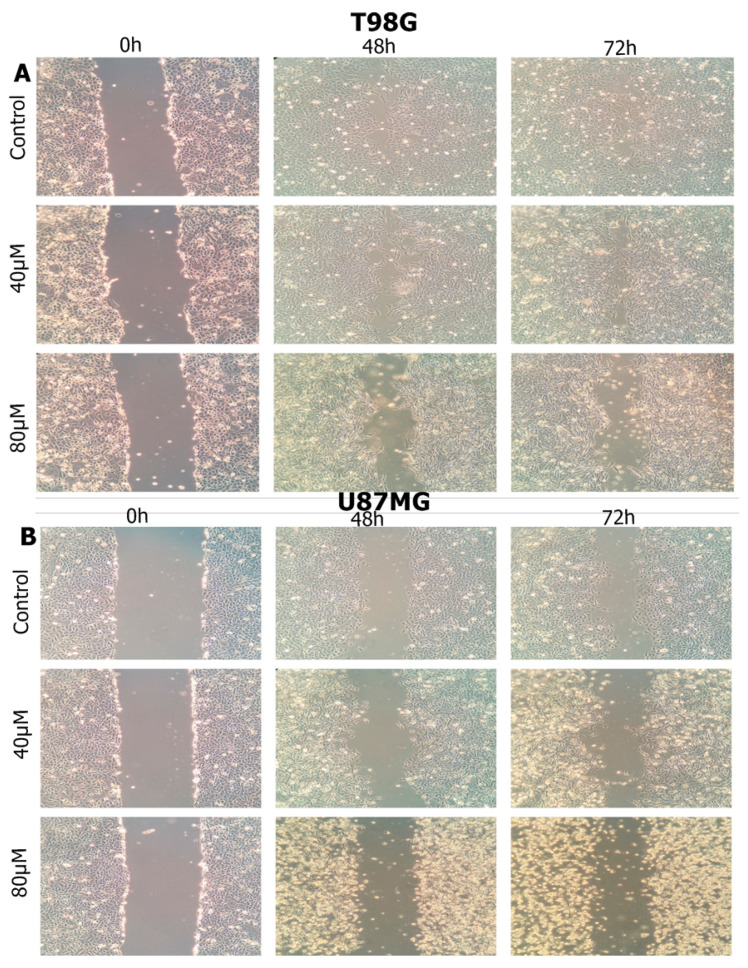
The influence of haloperidol on the migratory ability of glioblastoma cells at 48 and 72 h. (**A)**: Τ98, (**B**): U87, (×40). Approximately 100,000 cells were seeded in 6-well plates; when the cells grew to 70–80% confluence, they were exposed to concentrations of IC50 and twice the IC50 value of haloperidol. The monolayer cells were scratched using a 200 μL pipette tip at the bottom of the well and then cultivated under normal conditions.

**Figure 6:**
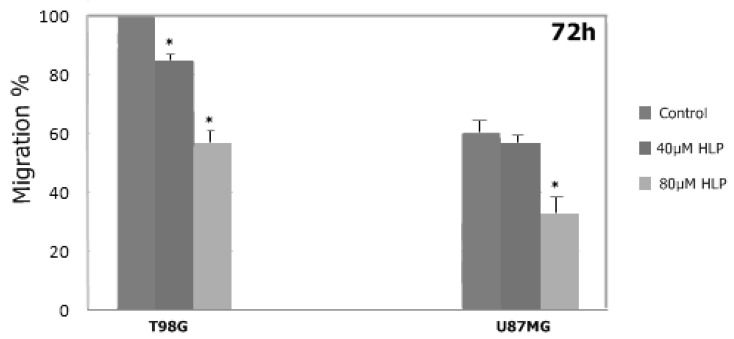
The influence of haloperidol (HLP) on the migratory ability of glioblastoma cells T98 and U87 at 72 h (quantified). Width_migration_ = Width_0h_ − Width_72h_ The experiment was performed in triplicate. Values expressed as migration percentages, and the wound widths at 0 h were set as 0%. Significant differences (*p* < 0.05) were marked with an asterisk.

To investigate whether haloperidol could affect the migration of U87 and T98 glioma cells into a wound generated by scratching, a cell monolayer showed that, at concentrations of 20 and 40 μM for U87 cells, and at 40 and 80 μM for T98 cells, haloperidol significantly inhibited the wound recovery of both cell lines (Figure 5; Figure 6).

### 3.6. Combination Treatment of Haloperidol, TMZ and Radiation Increased Cell Death

**Figure 7:**
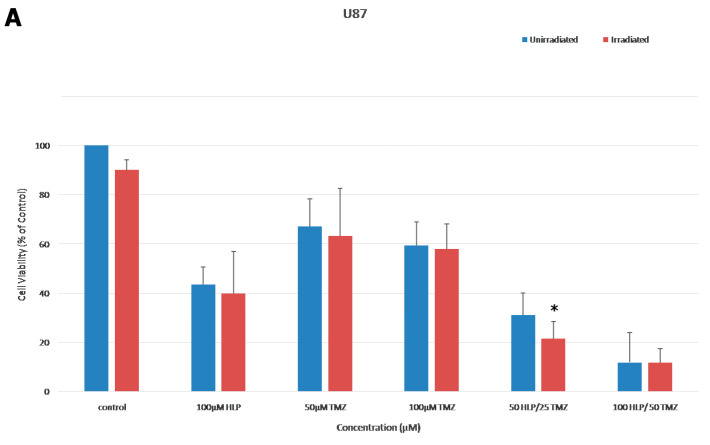

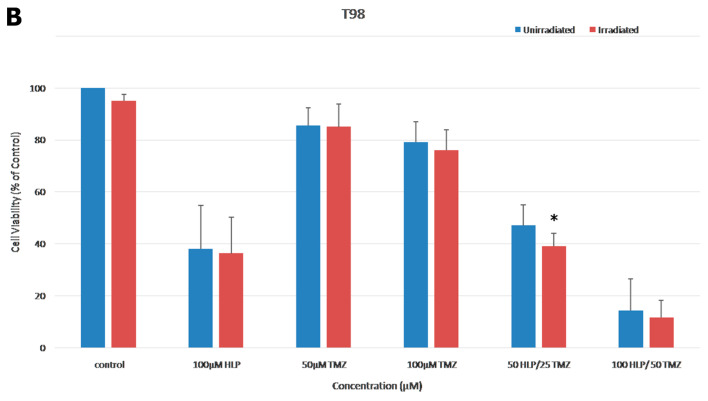
Combination treatment with haloperidol, TMZ and radiation. (**A**,**B**) 3-(4,5-Dimethylthiazol-2-yl)-2,5-diphenyltetrazolium bromide (MTT) assay on U87 (**A**) and T98 (**B**) GBM cell lines treated with haloperidol (HLP), temozolomide (TMZ), radiotherapy and their combination. Cell viability was assessed with the MTT assay (% control). Approximately 5000 cells were seeded in 96-well plates and after 24 h exposed to various concentrations of HLP alone or in combination with TMZ with and without radiation (4 Gy) and incubated for another 72 h. At 72 h, MTT was added and the amount of MTT-formazan was determined by 570 nm absorbance as the wavelength reference. Significant differences (*p* < 0.05) are marked with an asterisk.

To determine the effect of ionizing radiation in combination with haloperidol and/or TMZ, an escalating concentration of both drugs was administered to U87 and T98 cell lines. The IC50 values of TMZ were 50 μΜ in U87, and 160 μΜ in T98 cell lines. Cells were seeded and after 24 h were treated with haloperidol and/or TMZ, then 4 Gy radiotherapy was administered after a further 24 h. The MTT assay was conducted 24 h later. The effect of TMZ, as expected, was greater in U87 cell line. In both cell lines, the combination of haloperidol with TMZ and radiation therapy increased cell death (Figure 7).

## 4. Discussion

The present study demonstrated that haloperidol was an effective treatment for malignant glioma cells in vitro. Haloperidol induced the suppression of GBM cell growth, inhibited migration and produced cell cycle arrest in the G2/M phase. The induction of apoptosis was observed, with a significant decrease in the expression of CD44 and CD24 in the cells treated with haloperidol. The combination treatment of haloperidol, TMZ and radiation increased cell death. Cancer is the leading cause of death after cardiovascular disease. Although radiotherapy and chemotherapy have increased survival, the recurrence and side effects of treatment continue to limit their effectiveness, and new drugs that may improve current therapeutic options are urgently needed. To date, several studies have provided evidence that certain typical and atypical antipsychotic drugs may exert a chemotherapeutic effect in various forms of cancer [18,19]. Epidemiological studies have demonstrated a decreased incidence of several cancers in patients treated for schizophrenia, despite smoking and a lack of attention to exercise and other health-related behaviors in this population [20,21]. One study that included the records of 3226 patients with schizophrenia documented a significantly reduced risk of stomach, rectal and prostate cancer [21]. Furthermore, a recent meta-analysis showed an increased long-term mortality risk during follow-up in patients with schizophrenia who did not use antipsychotic drugs [22].

Haloperidol, a butyrophenone, is among the oldest antipsychotic drugs that are still used today. The main indications for its use include schizophrenia, delirium and bipolar disorder. This drug exerts its effect via strong antagonism, mainly of the D2 dopamine receptor. After the per os or intramuscular administration of haloperidol, the maximum concentration in plasma reaches 1–2 ng/mL and it is highly permeable in the central nervous system (CNS) [23]. The concentration of haloperidol in the rat’s brain was found to be 334.66 ± 63.01 ng/g, with a brain to plasma ratio of 18.16 [24]. Haloperidol has been shown to regulate the hedgehog signaling pathway by regulating 7-dehydrocholesterol reductase levels [19]. In addition, it has an antagonist effect on Sigma-1 receptors that have been implicated in vascular endothelial growth factor (VEGF) synthesis and proliferation [25,26]. Multidrug resistance (MDR) is one of the major mechanisms of cancer cells to evade treatment, operating with several mechanisms, such as decreased drug uptake and increased drug elimination. Several genes have been implicated in these processes, including the MDR1 gene that encodes the P-glycoprotein (P-gp) and that has been found in many cancers [27]. Several antipsychotic drugs, including haloperidol, have been reported to block the function of P-gp in vitro [28]. Furthermore, haloperidol has been shown to differentially affect the expression of arrestins and G protein-coupled receptor kinases (GRKSs) and ERK activity. Both GRKs and arrestins are the main regulators of G protein-coupled receptors (GPRC) signaling [29]. GPCRs have been implicated in many cell functions and in cancer cells on tumor growth, angiogenesis and metastasis [30,31].The effects of haloperidol on various different pathways make this drug an ideal candidate for drug repositioning.

GBM is the most common malignant primary brain tumor in adults. Current treatment involves gross total surgical excision, followed by chemoradiotherapy. In spite of aggressive treatment, recurrence is an inevitable event and mean survival is in the range of 12–15 months [1]. GBM is difficult to treat, and the BBB is an important contributor. Antipsychotics can penetrate the BBB; they have been studied extensively in other contexts, and thus time-consuming preclinical studies can be eliminated, and their side-effects are already known, which are usually related to dose levels and the potency of the drug [32]. In GBM, antipsychotics may exert their anti-malignant action via several pathways, such as the Wnt/β-Catenin signaling pathway, the epigenetic modification of histone deacetylase, and autophagy [33]. A recent study investigated the dopamine D2 receptor antagonists, haloperidol and risperidone, in glioma stem cells and two glioma cell lines (U251 and A172). An enhanced antiproliferative effect was found when the drugs were combined with TMZ, similar to the effect observed in our study. Treatment with TMZ upregulated the expression of dopamine D2 receptors; haloperidol and risperidone, however, it did not enhance the apoptosis induced by TMZ, but produced more autophagosomes [9]. In the present study we found that haloperidol, in addition, induced a G2/M cell cycle arrest and an increase in the subG0/G1. Drugs that produce G2/M arrest, which is the radiosensitive phase of the cell cycle, are potent radiosensitizers [34]. This applies to TMZ, which produced a pronounced G2/M cell cycle arrest [17]. The apoptotic effect may be connected with the transactivation of other molecular targets. The antiproliferative effect of haloperidol might not be connected with action on dopamine D2 receptors, since haloperidol IC50 on cell proliferation is much higher than its inhibition of D2 dopamine receptors (~ 40 µM vs. ~30 nM) and there is no significant change in many dopamine D2 receptors in GBM, compared to normal tissue, as depicted in The Cancer Genome Atlas (TCGA). Thus, systemic-low dose haloperidol application might provide significant anti-GBM action without systemic toxicity or psychiatric side-effects. Radiotherapy is currently the most effective treatment for GBM, but has only rarely been used in the investigation of combination treatments for GBM [35]. In the present study, we showed that haloperidol increased cell death when combined with radiation for GBM cells. Based on previous studies, for all experiments we used a dose of 4 Gy using X-rays generated by a linac 6 MV accelerator [15]. At this dose, G2/M arrest in T98 cells and S-phase arrest in U251 cells were observed [17]. In the treatment of GBM, the daily dose administered to patients is 2 Gy. The usual side effects of haloperidol are drowsiness and cognitive dysfunction.

We also investigated the expression of several CD markers, because they were implicated in cancer migration, invasion, and metastases, and they had not previously been studied in cancer cells treated with haloperidol. Haloperidol was associated with a significant decrease in CD24 and CD44, both of which have been related to cancer stem cells. CD24 positive tumors are reported to have a poor prognosis, and CD44 depletion has been shown to inhibit tumor proliferation and augment the effect of cytotoxic drugs [36,37]. Valproic acid, an effective antiepileptic drug, exerts a potential anti-GBM effect via the inhibition of angiogenesis and the downregulation of 0-6-methylguanine-DNA methyltransferase (MGMT) expression, which has been found to decrease CD44 expression in several glioma cell lines [38]. Thus, decrease in CD24/CD44 expression could be a novel mechanism of the antineoplastic action of haloperidol. Another important finding of the present study was the inhibitory effect of haloperidol in the migration of GBM cells in a wound healing assay. Other antipsychotics, such as sertindol and trifluoperazine, have also been shown to inhibit migration [39,40], but further experiments, with an additional invasion assay, are needed to confirm this observation.

The present study has several limitations. Plasma levels of haloperidol in patients with schizophrenia range from a low of 2 to 13 ng/mL to a high of 24.1 to 35 ng/mL [41]. The dose of 100 μM that it was used in some of our experiments and is therefore considered high. Thus, ways should be investigated for delivering high doses of the drug to the tumor without severe systemic side effects. Encapsulation into liposomes or other carriers might be of interest as drug delivery options, or combination strategies with other available drugs or other antipsychotics. Secondly, evaluating the effect of radiation 3 days after the treatment might be preliminary and might need further experiments using additional methods, such as a colony-forming assay, where the effect on cell viability is more obviously observed 10–14 days after irradiation [42]. Thus, further, longer experiments are needed to explore whether haloperidol combined with irradiation enhances GBM cell death. Thirdly, the intracellular pathways mediating haloperidol action need to be further clarified. Differences in the GBM cell response to haloperidol might be related to the genetic heterogeneity presented by different cell lines derived from GBM.

Overall, given that GBM is a tumor that is difficult to treat, with the additional obstacle for many agents of the BBB, haloperidol appears to offer potential as an innovative treatment option. In view of the difficulty of finding novel anticancer drugs, drug repurposing constitutes a promising approach, given the known safety profile and the consequent diminished cost of phase I–II studies [43]. There is definitely a need for further studies to better elucidate the mechanism of haloperidol action in cancer, and to validate our results in patient-derived cell lines, glioma xenograft models and clinical trials.

## Figures and Tables

**Table 1:** Cell-cycle distribution assessed by flow cytometry in U87 glioblastoma cells. Haloperidol induced G2/M cell cycle arrest.

Treatment	SubG0 (M4)	G0/G1 (M1)	S (M2)	G2/M (M3)
Control	1.07 ± 0.4	80.56 ± 4.3	8.17 ± 2.6	10.72 ± 2.2
20 μM haloperidol	1.88 ± 0.5	69.2 ± 3.1	7.37 ± 1.2	21.15 ± 4.7
40 μM haloperidol	2.29 ± 0.8	51.92 ± 3.5	10.73 ± 3.1	35.02 ± 2.5
50 μM haloperidol	6.83 ± 1.2	48.59 ± 4.2	12.54 ± 2.9	32.89 ± 5.7

## Supplementary Materials

The following are available online at https://www.mdpi.com/2227-9059/8/12/595/s1.
