# Supplementary material for: Haloperidol Induced Cell Cycle Arrest and Apoptosis in Glioblastoma Cells
Source: Biomedicines. 2020 Dec 11;8(12):595. doi: 10.3390/biomedicines8120595 (PMC7763579; doi:10.3390/biomedicines8120595)
Supplement: Supplementary file 1 [file biomedicines-08-00595-s001.pdf]

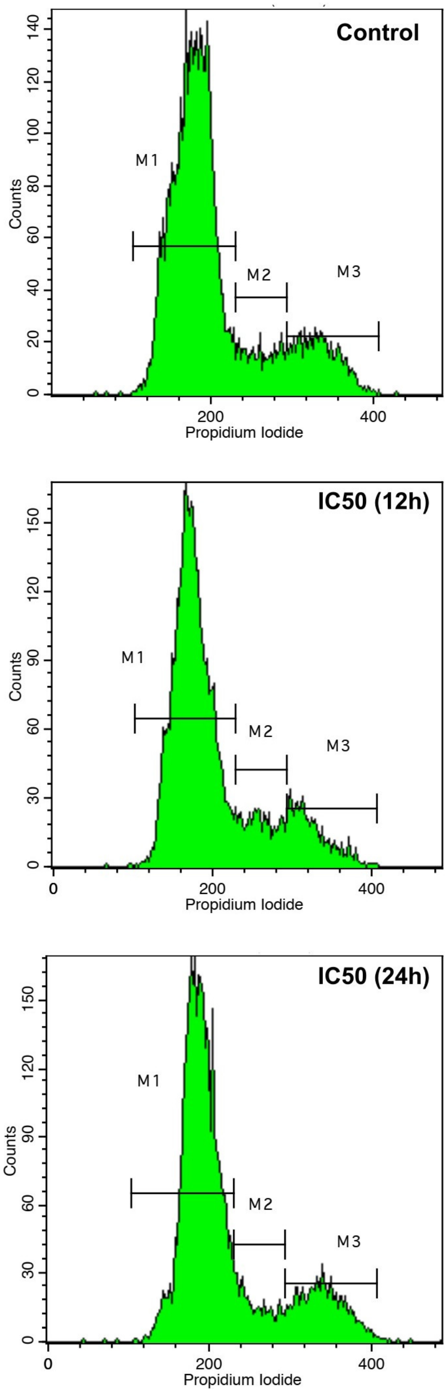

Marker	% Gated
All	100.00
M1	78.58
M2	8.93
M3	12.69

Marker	% Gated
All	100.00
M1	77.81
M2	11.34
M3	11.27

Marker	% Gated
All	100.00
M1	75.43
M2	9.11
M3	15.75

Figure S1. The effect of haloperidol on DNA content and cell cycle distribution of U87 after 12h and 24h treatment. Cells were incubated for 12h or 24h with haloperidol at IC50 values and DNA content was assessed immediately, after propidium iodide staining, using flow cytometry.
